# Supplementary figures and images for: Influence of glycoprotein MUC1 on trafficking of the Ca2+-selective ion channels, TRPV5 and TRPV6, and on in vivo calcium homeostasis
Source: J Biol Chem. 2023 Jan 20;299(3):102925. doi: 10.1016/j.jbc.2023.102925 (PMC9996365; doi:10.1016/j.jbc.2023.102925)

# Supporting Information

## Figure S-1

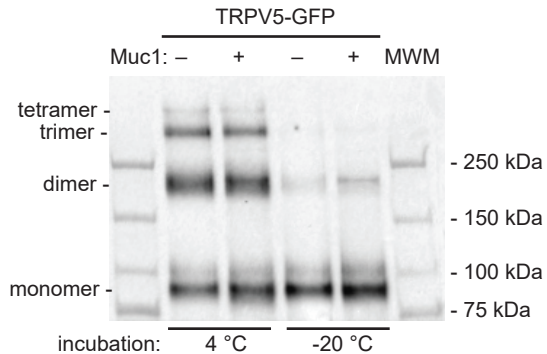

Supplement: Supporting Figure S1 — Incubation of cell lysates overnight with neutravidin beads results in oligomerization of TRPV5-GFP. A control experiment is shown comparing immunoblot of cell extract with anti-GFP antibody after incubation overnight at 4 °C versus −20 °C, demonstrating that differences in stoichiometry were the result of the overnight incubation and not indicative of differences in stoichiometry in cells. Therefore, all stoichiometric bands were included for quantification of TRPV5-GFP in Figure 1. [file mmc1.pdf]
